# 

**Published:** 1918

**Authors:** 


PUBLISHED MONTHLY. — ATLANTA — SUBSCRIPTION $1.00

### JOURNAL-RECORD OF MEDICINE

SURGEON GENERAL'S OFFICE

Successor to { Atlanta Medical and Surgical Journal, Established 1855. and Southern Medical Record, Established 1870. } 64th Year

Vol. LXIV — Atlanta, Ga., Mar-Apr. 1918 — No. 12

